# Two High-Precision Proximity Capacitance CMOS Image Sensors with Large Format and High Resolution †

**DOI:** 10.3390/s22072770

**Published:** 2022-04-04

**Authors:** Yuki Sugama, Yoshiaki Watanabe, Rihito Kuroda, Masahiro Yamamoto, Tetsuya Goto, Toshiro Yasuda, Hiroshi Hamori, Naoya Kuriyama, Shigetoshi Sugawa

**Affiliations:** 1 Graduate School of Engineering, Tohoku University, 6-6-11-811, Aza-Aoba, Aramaki, Aoba-ku, Sendai 980-8579, Miyagi, Japan; yoshiaki.watanabe.q2@dc.tohoku.ac.jp (Y.W.); masahiro.c.yamamoto@sony.com (M.Y.); 2 New Industry Creation Hatchery Center, Tohoku University, 6-6-10, Aza-Aoba, Aramaki, Aoba-ku, Sendai 980-8579, Miyagi, Japan; tetsuya.goto.b2@tohoku.ac.jp (T.G.); shigetoshi.sugawa.d4@tohoku.ac.jp (S.S.); 3 OHT Inc., 1118-1, Nishinakajo, Kannabe-cho, Fukuyama 720-2103, Hiroshima, Japan; to_yasuda@ohtinc.jp (T.Y.); hi_hamori@ohtinc.jp (H.H.); 4 LAPIS Semiconductor Co., Ltd., 2-4-8, Shin-Yokohama, Kohoku-ku, Yokohama 222-8575, Kanagawa, Japan; kuriyama787@lapis-semi.com

**Keywords:** CMOS, proximity capacitance, image sensor, high precision, large format, high resolution

## Abstract

This paper presents newly developed two high-precision CMOS proximity capacitance image sensors: Chip A with 12 μm pitch pixels with a large detection area of 1.68 cm^2^; Chip B with 2.8 μm pitch 1.8 M pixels for a higher resolution. Both fabricated chips achieved a capacitance detection precision of less than 100 zF (10^−19^ F) at an input voltage of 20 V and less than 10 zF (10^−20^ F) at 300 V due to the noise cancelling technique. Furthermore, by using multiple input pulse amplitudes, a capacitance detection dynamic range of up to 123 dB was achieved. The spatial resolution improvement was confirmed by the experimentally obtained modulation transfer function for Chip B with various line and space pattens. The examples of capacitance imaging using the fabricated chips were also demonstrated.

## 1. Introduction

Image sensors can capture two-dimensional distribution of physical quantities in the real world. There are various types of image sensors depending on the information to be acquired, such as optical image sensors that detect the intensity of light, and ToF image sensors that detect distance based on time differences. These image sensors are used not only in digital cameras for ornamental photography, but also in various fields to improve safety, security, and productivity, such as in-vehicle cameras for autonomous driving and machine vision cameras for factory automation [1,2,3].

Capacitance sensors can nondestructively measure the capacitance between a sensor and a target. By measuring the capacitance, it is possible to detect the presence or absence of an object near the sensor and the distance between the object and the sensor. It is used in noncontact switches, level sensors, continuous cell density measurement devices [4], and pressure sensors [5].

A proximity capacitance image sensor, which consists of an array of capacitance sensors, can detect and visualize two-dimensional distributions of capacitance between sensor and target. Unlike optical image sensors, these sensors can detect electrical connections as well as microstructures and minute irregularities on the surface of objects and distribution of substances inside organic, solid, and liquid materials. Because of these features, they are used in various applications such as wiring inspection for flat panel displays and printed circuit boards [6,7,8], fingerprint authentication [9,10,11,12], observation of cellular reaction processes [13,14], and so on. These applications require high detection precision of aF (10^−18^ F) or lower, as well as the ability to capture a sufficiently large area and a clear image of minute targets. For example, in fingerprint authentication, an area of about 1 cm^2^ is required to capture the entire fingerprint, and in wiring inspection, it is desirable to have as large a detection area as possible to efficiently inspect the entire large area of the substrate. In addition, a resolution on the order of µm is necessary for wiring inspection of high-resolution displays with fine wiring and for cell observation. Furthermore, it is desirable to have a wide dynamic range performance that can capture a wide range of capacitance in a single image.

There are several methods of detecting capacitance, such as those that convert capacitance to frequency [13], current [9,10,11], or voltage [6]. In the method of converting the capacitance to frequency or current, it is difficult to reduce the pixel pitch and to detect the capacitance with high precision due to its circuit configuration. Therefore, in this work, we adopted the method of converting to voltage, which has a simple circuit structure, to realize high-precision detection in minute pixels.

Previous works have reported discrete sensors with aF-order detection precision [13,15,16] and an array sensor with a detection area of 8.73 cm^2^ with 11.4 µm pitch pixels [6]. Previously, we have presented a prototype CMOS proximity capacitance image sensor with 256^H^ × 256^V^ 16 µm pitch pixels achieving 100 zF detection precision due to an advanced noise cancelling technique [17,18,19,20,21,22]. However, the simultaneous achievement of a detection precision of less than aF and a detection area of more than 1 cm^2^, or a pixel pitch of less than 10 μm toward a higher resolution, has not been reported yet.

In the paper of IISW 2021 [23], we presented two newly developed chips for increasing imaging area and spatial resolution: Chip A with large format 12 µm pixels for practical inspection applications and Chip B with high-resolution 2.8 µm pitch pixels. Large-area and high-resolution capacitance detection with a precision of less than 1 aF by using these two chips has been demonstrated. In this paper, we additionally describe the more detailed design and characterization results, calculation and verification results of the detection precision, and the measurement results of the resolution using the modulation transfer function analysis. Furthermore, examples of images for wiring inspection applications will be presented.

## 2. Design and Structure of Developed Chips

**Figure 1:**
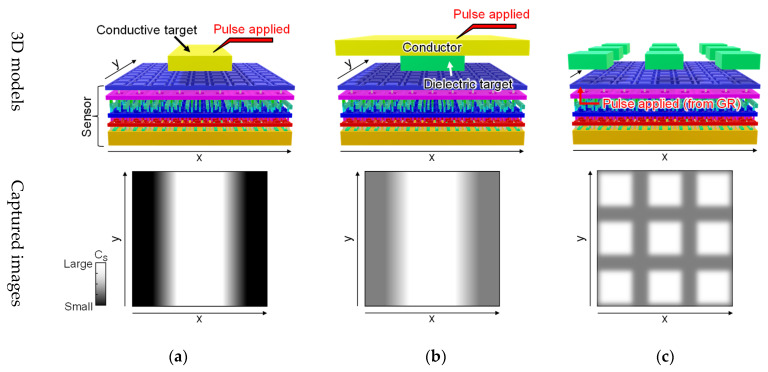
The 3D models of the sensor and the target, and the captured images for each target: (**a**) conductive target; (**b**) dielectric target; (**c**) particulate targets.

Figure 1 shows 3D models illustrating the proximity capacitance imaging setup and the images captured for each target. For the proposed proximity capacitance sensors, a counter electrode was introduced to which the input pulse signal was supplied. For a conductor target, the input pulse signal was supplied by a probe and the target itself was used as the counter electrode, as in Figure 1a. It was also possible to measure proximity capacitance without a probe by supplying the input pulse signal through a coupling capacitance between the target and the counter electrode in the chip. For a dielectric target, the measurement was performed by placing a flat counter electrode and supplying an input pulse signal to it, as in Figure 1b. For particle targets, measurement was performed by using the guard ring in the sensor as a counter electrode and supplying the input pulse signal to it, as in Figure 1c. By using an appropriate measurement method, various targets could be measured.

**Figure 2:**
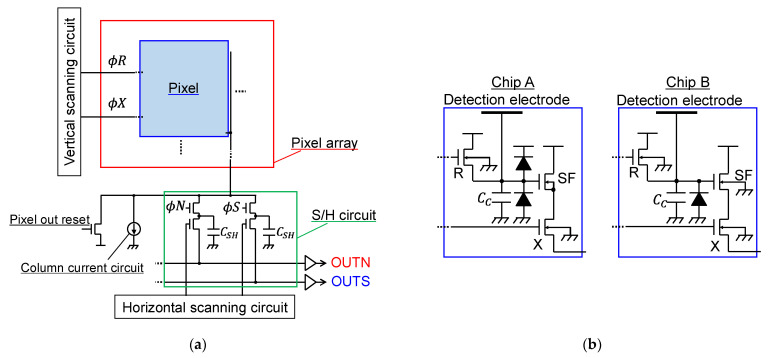
The schematics of the two chips: (**a**) the circuit block diagram; (**b**) the circuit schematic of the pixels.

Figure 2 shows the circuit block diagram of the developed chips. A schematic of the overall circuit is shown in Figure 2a, which employed the rolling shutter method used in general CMOS image sensors. When capturing an image, the vertical scanning circuit first selected a row to acquire the signal for one row, then, the column S/H circuit held the signal, and the horizontal scanning circuit selected a column to read the analog signal out of chip in turn. Here, the signal was readout from the column S/H circuit to the horizontal signal readout lines by capacitive charge division operation. The analog output signal was digitized by on-board ADC with 20 MHz signal sampling rate. This was repeated for all the rows to obtain the signals for one frame. In this way, high-speed and low-noise readout was achieved. As shown in Figure 2b, the pixel circuits were equipped with a capacitance detection circuit using a capacitance–voltage conversion method, and consisted of a detection electrode, a reset transistor (R), an SF transistor (SF), a select transistor (X), a protection diode, and detection electrode parasitic capacitance (C_C_). Chip A used an isolated P-well in a deep N-well for the SF transistor, which was to improve the gain and extend the linear range of the SF by eliminating the substrate bias effect. It also allowed the formation of a PN protection diode for the high-voltage side. Chip B, on the other hand, used a normal SF transistor structure and removed the protection diode for the high-voltage side to reduce the layout area and greatly reduce the pixel pitch. Furthermore, by using only one type of protection diode, it was possible to calculate C_C_ from the photon transfer curve using the parasitic light sensitivity of the protection diode [24].

**Figure 3:**
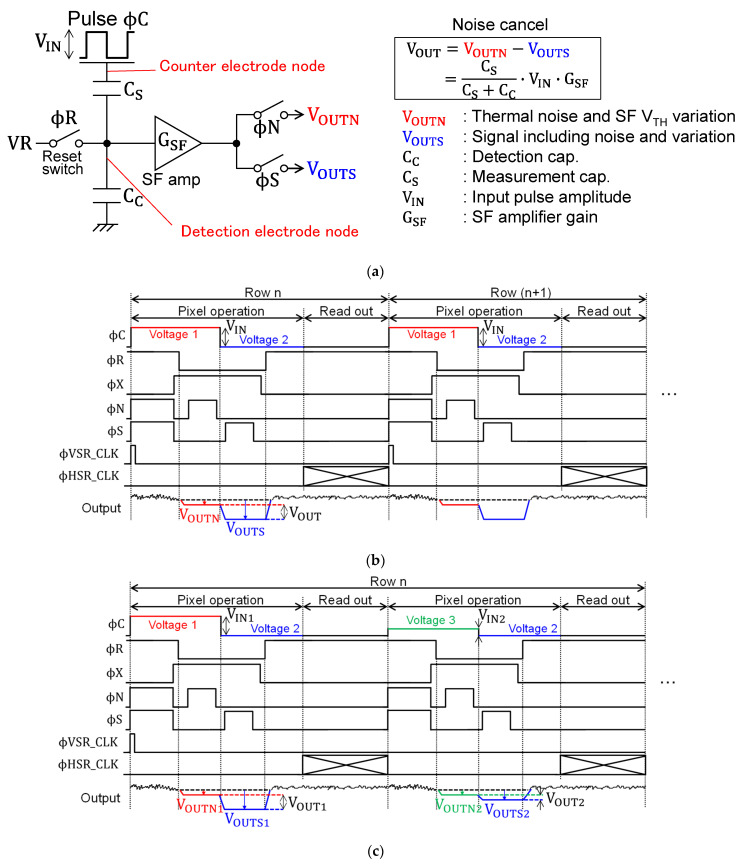
Principle of operation of proximity capacitance image sensor: (**a**) simplified circuit and noise cancelling principle; (**b**) timing diagram of normal mode; (**c**) timing diagram of HDR mode.

Figure 3 shows a simplified sensor circuit and operational timing diagrams of the normal and the high dynamic range (HDR) modes. As shown in Figure 3a, each pixel contained detection electrode capacitance (C_C_) which was connected to the measurement capacitance (C_S_) in series. When detecting the capacitance, the detection electrode node in the pixel was reset by the reset switch, φC was applied, and the voltage of the floating detection electrode node was read out by the SF amplifier. After resetting and turning off the reset switch, thermal noise remained at the detection electrode node. It was due to the thermal fluctuations of the charge, and it was random in time. Therefore, every time the reset switch was turned off, the voltage at the detection electrode node was changed, which appeared as temporal random noise (RN) in the output signal without noise cancelling. Furthermore, since the threshold voltage of the SF transistor used for readout varies from pixel to pixel, the gain and offset values also varied from pixel to pixel. This was to appear as a fixed pattern noise in the image without noise cancelling. These noises degraded the precision of capacitance detection.

In this work, a noise-cancelling technique was introduced to reduce these noises and achieved high-precision capacitance detection. Figure 3b shows the pulse timing diagram in the normal mode with the noise cancelling technique applied. First, the counter electrode was set to the first voltage by φC, and φR was turned on to reset the detection electrode node. Then, when φR was turned off, thermal noise remained in the detection electrode node. At this time, the voltage of the detection electrode node was read out by the SF amplifier with φN turned on and was designated as V_OUTN_. After that, when the counter electrode was changed to the second voltage level, the voltage of the detection electrode in the floating state changed according to the ratio of C_C_ and C_S_ and the input pulse amplitude V_IN_. Then, φS was turned on and read out, the voltage of the detection electrode node used the same SF amplifier as the S signal, and the result was V_OUTS_. V_OUTN_ included noise components such as thermal noise and V_TH_ variation, and V_OUTS_ included signal components superimposed on them. Finally, by taking the difference between V_OUTN_ and V_OUTS_, only the signal component with noise component removed could be obtained. The output V_OUT_ was expressed by the following Equation (1).

(1) $$V_{\mathrm{OUT}}=V_{\mathrm{OUTN}}-V_{\mathrm{OUTS}}=\frac{C_{S}}{C_{C}+C_{S}}\times V_{\mathrm{IN}}\cdot G_{\mathrm{SF}}$$

Here, the proximity capacitance signal (V_OUT_) is proportional to the voltage amplitude (V_IN_) applied to the counter electrode. For the chips presented in the paper, V_OUTN_ and V_OUTS_ were readout to the column S/H circuit during the pixel operation period, then, these signals were readout in parallel using the two horizontal signal lines and output buffers as described in Figure 2a. An on-board differential ADC was employed to achieve signal subtraction of V_OUTN_ and V_OUTS_ and digitize the V_OUT_. Since V_OUTN_ and V_OUTS_ were output in parallel, the noise canceling did not impact the horizontal signal readout period. The additional operation time by the introduced noise canceling was only the signal readout time of V_OUTN_ during the pixel operation period, and it was about eight microseconds or fewer per one row. The larger the applied V_IN_, the smaller the C_S_ detected, but if the C_S_ was large, the signal output would saturate beyond the signal readout range of the chips. In HDR mode, as shown in Figure 3c, the third voltage was used in addition to the first and second voltages, and two types of signals could be obtained by applying large and small V_IN_ to each row. This made it possible to capture the region with small C_S_ and large C_S_ simultaneously. Therefore, it was possible to expand the dynamic range of the detection capacitance compared to that of the normal mode with one type of V_IN_.

**Figure 4:**
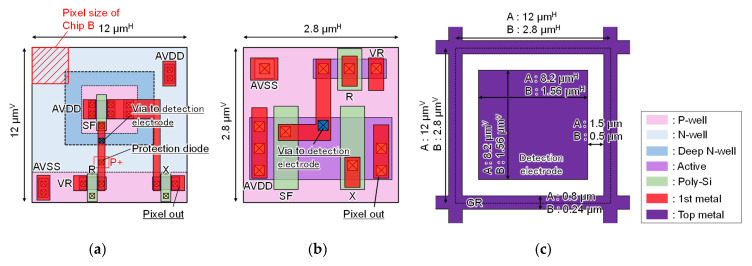
The pixel layout diagrams of the two chips: (**a**) up to the 1st metal layer of Chip A; (**b**) up to the 1st metal layer of Chip B; (**c**) the top metal layer.

Figure 4 shows the layout diagrams of the developed pixels, Figure 4a for Chip A and Figure 4b for Chip B, up to the first metal layer. The pixel of Chip A had a 12 μm pitch, a well-in-well structure for the SF transistor, and an N^+^P protection diode. The pixel of Chip B had a pitch of 2.8 μm and used a standard p-well structure for the SF transistor. In addition, in both pixels, the source of the reset transistor was used as an N^+^P protection diode. Figure 4c shows the top metal layout of both chips. The center was the detection electrode, which was connected to the gate of the SF transistor on the lower layer through vias. Typically, the guard ring around the detection electrode was connected to the GND, which suppressed the crosstalk of the electric field between adjacent pixels and improved the resolution. It was also possible to detect capacitance by applying φC directly from the guard ring without using an external probe. Additionally, strict light shielding was used to suppress the light effect during capacitance detection. The value of Cc was determined by the parasitic capacitance with respect to the detection electrode. Thus, control of the parasitic capacitance in the pixel was very important in the developed sensors. In order to minimize Cc to improve the sensitivity, the metal wiring layout was carefully designed to reduce the parasitic capacitance.

## 3. Chip Fabrication and Measurement Results

**Figure 5:**
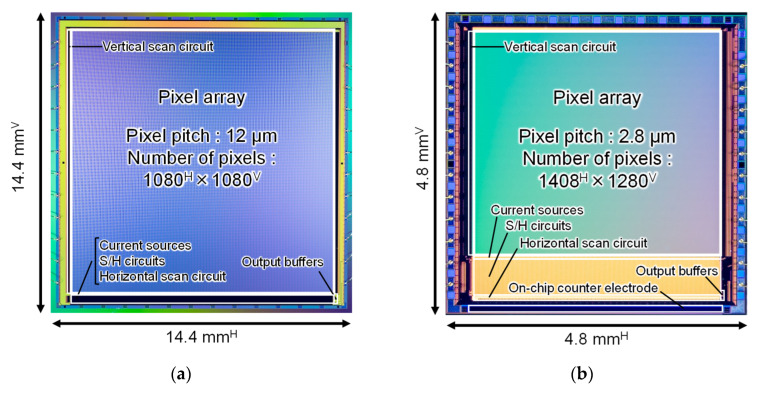
The micrograph of the fabricated CMOS proximity capacitance image sensors: (**a**) Chip A; (**b**) Chip B.

Figure 5 shows the micrographs of the fabricated chips. A 0.18 µm 1-poly-Si 5-metal CIS technology was employed. The chip size, number of pixels, and pixel pitch were 14.4 × 14.4 mm^2^, 1080^H^ × 1080^V^, and 12 µm for Chip A, and 4.8 × 4.8 mm^2^, 1408^H^ × 1280^V^, and 2.8 µm for Chip B, respectively. Chip B had an on-chip counter electrode to apply φC through the coupling capacitance to the conductive target.

**Figure 6:**
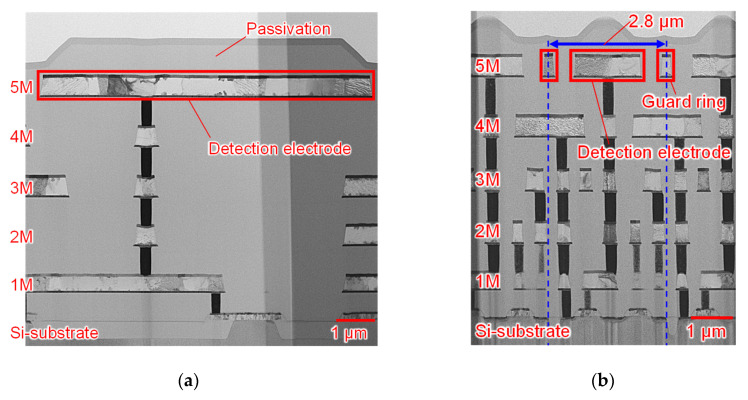
The cross-sectional pixel TEM image of the two chips: (**a**) Chip A; (**b**) Chip B.

Figure 6 shows the cross-sectional pixel TEM images of the two chips. The first through the fourth metal layer was used for wiring and light shielding, and the fifth metal on the top layer was used as a detection electrode and guard ring. The detection electrode was protected by a passivation film, and the detection electrode and transistor were connected through vias.

**Figure 7:**
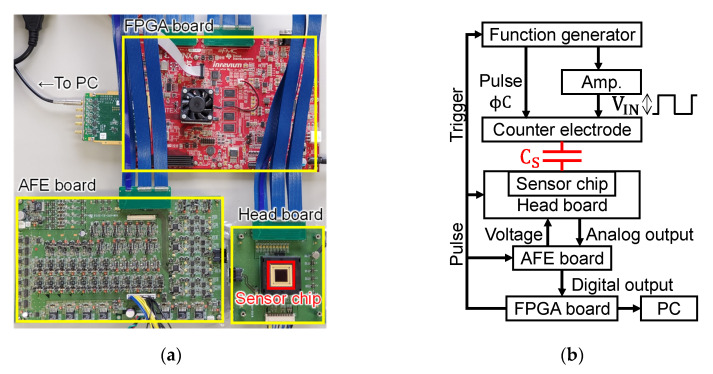
The measurement system for characterizing the performance of fabricated sensor chips: (**a**) picture; (**b**) block diagram.

Figure 7 shows the measurement system. It consisted of a headboard with a fabricated sensor chip mounted face-up, an analog front-end (AFE) circuit board with voltage regulators and a 14-bit differential ADC directly connected to V_OUTN_ and V_OUTS_, a FPGA board to supply operation pulses to the sensor chip, and a PC. A function generator was used to apply φC to the counter electrode, and a trigger signal was input from the FPGA to synchronize with the sensor operation. The function generator alone could generate φC up to V_IN_ = 20 V, and the sensor was measured at a maximum of V_IN_ = 300 V using a high-speed high-voltage amplifier.

**Figure 8:**
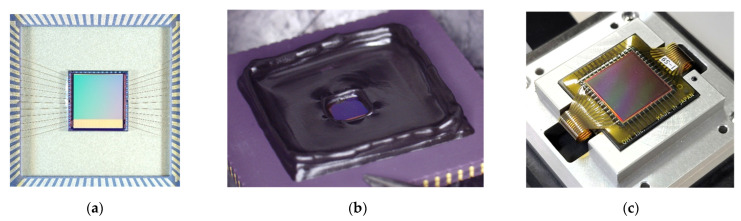
Various assembly methods of the sensor chips: (**a**) ceramic package with bonding wires; (**b**) resin potting; (**c**) polyimide film with flexible wires.

Figure 8 shows examples of chip assembly methods: Figure 8a is the assembly into a ceramic package using bonding wires. This was the simplest assembly method, and the capacitance was detected by placing a target directly in proximity to the sensor surface. In Figure 8b, the chip in Figure 8a, except for the pixel area, was encapsulated with a thermoplastic resin. The resin protected bonding wires and created a bank outside the pixel area of the chip, which enabled capacitance detection of liquid or particulate targets. Image Figure 8c shows the assembly using a polyimide film and flexible wiring. In this method, the chip surface and wiring were protected, and the sensor surface could be made flat, so that capacitance could be detected for large-area targets such as fingerprints and flat-panel displays.

**Figure 9:**
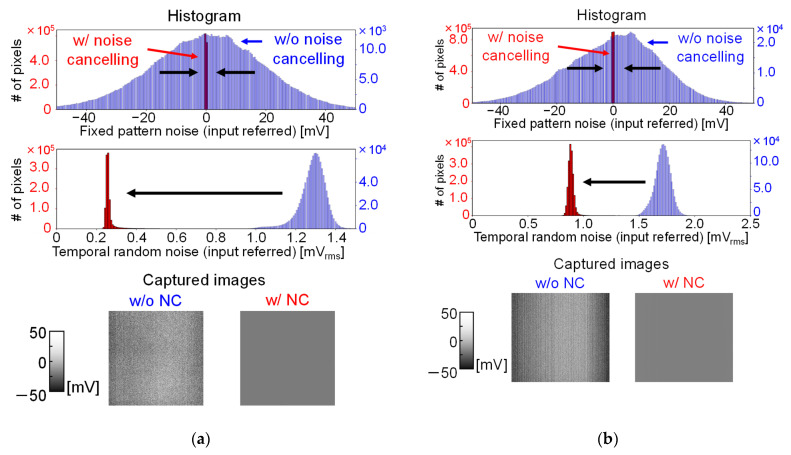
Captured images without and with noise cancelling and their histograms in the absence of targets: (**a**) Chip A; (**b**) Chip B.

Figure 9 and Table 1 show the measured noise characteristics without any measurement targets obtained without and with the noise cancelling. Without the noise cancelling, the input referred fixed pattern noise and temporal random noise arose mainly due to the threshold voltage variation of SF and column fixed pattern noise, and the kTC noise at the detection electrode node. Without noise cancelling and averaging, V_FPN_ and V_RN_ were 19.3 and 1.29 mV_rms_, respectively for Chip A and 16.4 and 1.71 mV_rms_ for Chip B, respectively. For Chip B, the histogram of the FPN was not symmetrical. It was considered that this asymmetrical noise histogram was due to the column FPN and shading appearing in the image without noise canceling. We considered that the cause of the column FPN and the shading were due to the IR drop of the VSS node, which induced the variation of the current for the column current sources. When noise cancelling technique was applied here, the noise was significantly reduced to 37.8 and 267 µV_rms_ for Chip A and 137 and 887 µV_rms_ for Chip B, respectively. The column FPN and shading were also cancelled in this readout operation. Furthermore, by averaging multiple frames, the RN decreased in inverse proportion to the square root of the number of averaged frames and could be reduced to about 1/10 when 100 frames were averaged. We thus achieved high detection precision with the noise cancelling technique and confirmed further precision improvement by averaging multiple frames.

**Figure 10:**
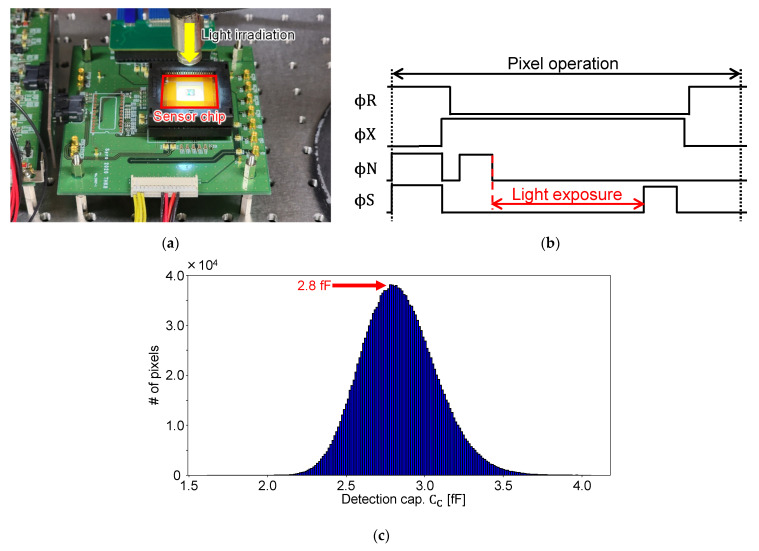
Measurement to determine the capacitance value of C_C_: (**a**) sensor chip irradiated with light; (**b**) pulse timing; (**c**) histogram of measured C_C_ of Chip B and C_C_ values of the two chips.

In the proximity capacitance image sensor of this work, an input–output relation expressed in Equation (1) indicated that the capacitance value of C_C_ must be obtained to calculate C_S_. Therefore, two methods were used to calculate the capacitance value of C_C_: one by simulation and the other by measurement using the fabricated chip. In the first method, for both chips, the parasitic wire capacitance of the wiring was extracted from the post-layout simulation, and the parasitic capacitance of the transistors was calculated from AC analysis results. In the second method, the photoelectric conversion characteristics were measured from the parasitic light sensitivity in the protection diodes in the pixels using the fabricated chip, and the capacitance value was calculated from the photon transfer curve (PTC). The calculation from this measurement could be done only with Chip B, which had only one protection diode type. We utilized this protection diode as a pixel photodiode. This measurement method is shown in Figure 10a,b. The measurement was performed by irradiating a high-intensity light from near the front of the chip, as shown in Figure 10a. Since the diffusion layer in the pixel was shielded by the metal wiring layer, no optical signal could be obtained when it was operated normally as a capacitance sensor. However, as shown in Figure 10b, by expanding the exposure time significantly and the time between N and S sampling, it was possible to obtain an optical signal sufficient for measurement from the parasitic light sensitivity. Image Figure 10c shows the histogram of C_C_ for Chip B obtained by the measurement and the values of C_C_ for both chips. During this experiment, no counter electrode signal was provided, and we assumed that the value of Cs was negligibly small in comparison to Cc. From the measurement, we obtained a C_C_ of 2.8 fF for Chip B, which was close to the extracted value of 2.5 fF. The extracted value by the simulation agreed well with the measured one, indicating the parasitic capacitance extraction was accurately conducted. For Chip A, the value of 5.4 fF was obtained from the simulation.

**Figure 11:**
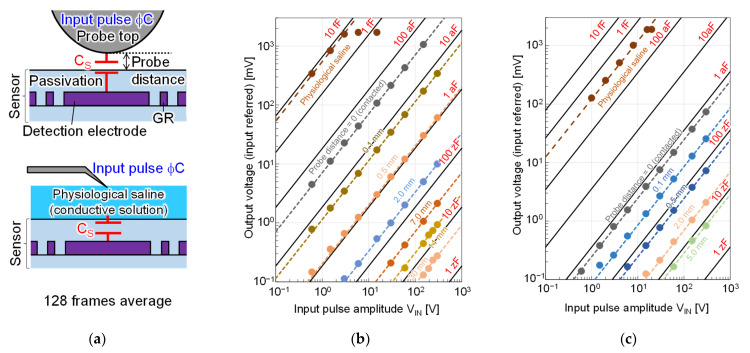
Measured transfer characteristics of the two chips: (**a**) measurement methods; (**b**) Chip A; (**c**) Chip B.

Figure 11 shows the measured transfer characteristic of the two chips with various measurement capacitance conditions. As shown in Figure 11a, the measurements were performed by changing the distance between the chip surface and the probe, which was the counter electrode, or by dropping saline, which was a conductor, on the chip surface. Image Figure 11b shows the measurement results of Chip A, and Figure 11c shows the measurement results of Chip B. Here, the signal output was plotted as a function of the voltage amplitude of the input pulse. Colored dots show the measured values, and the black lines are the calculated characteristics at each capacitance value, using Equation (1) and the C_C_ obtained from the simulation and the measurement for the Chips A and B, respectively. The measurement results show that the input referred signal range was confirmed to be over 1.0 V with good linearity for both chips. Chip A achieved a lower limit of detection capacitance of 70 zF when V_IN_ was 20 V, which was confirmed to be safe when applied with a target in contact with the chip surface or applied to the human body, and 5 zF when V_IN_ was 300 V, which was the maximum voltage that can be applied in the measurement system used. By adjusting V_IN_ in the range below 300 V, a wide dynamic range of 123 dB from 5 zF to 6.9 fF, which is the limit determined by the structure, was shown. The highest SNR was 76 dB. Similarly, Chip B was shown to achieve a lower limit of detection capacitance of 100 zF at V_IN_ of 20 V and 8 zF at 300 V, with a dynamic range of 94 dB from 8 zF to 0.4 fF and a maximum SNR of 61 dB.

**Figure 12:**
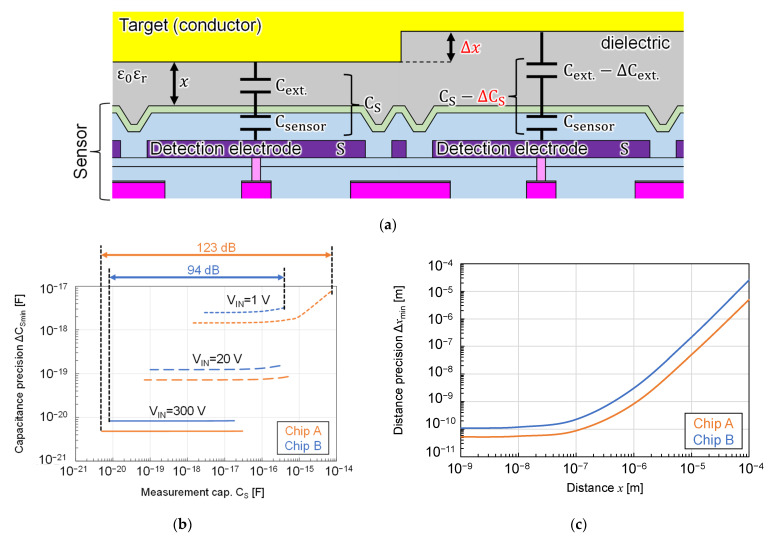
Calculation of capacitance and distance detection precision: (**a**) cross-sectional schematic diagram of a chip surface and a conductor target with small irregularities; (**b**) calculation results of the relation between the measurement capacitance C_S_ and the capacitance detection precision ΔC_Smin_ at each input voltage V_IN_; (**c**) relation between the reference distance x between the chip surface and the target and the distance detection precision Δx_min_.

The detection precision of the sensors was calculated from the noise and C_C_ values and transfer characteristics obtained so far. Figure 12a shows the cross-sectional diagram of the chip surface and the conductive target. Assume that a capacitance C_S_ was formed between the detection electrode and the target when the distance between the chip surface and the target was x. When C_S_ became smaller by ΔC_S_, the minimum ΔC_S_ that could detect this small change was determined by the capacitance detection precision ΔC_Smin_. This ΔC_Smin_ can be expressed as the following Equation (2). It assumed that the capacitance could be measured when its signal was larger or equal to the noise level.

(2) $${\Delta C}_{\mathrm{Smin}}=\frac{V_{\mathrm{RN}}{\left(C_{C}+C_{S}\right)}^{2}}{V_{\mathrm{RN}}\left(C_{C}+C_{S}\right)+C_{C}V_{\mathrm{IN}}}$$

From this equation, we can see that ΔC_Smin_ varies with ΔC_S_. Image Figure 12b shows the relation between C_S_ and ΔC_Smin_ at each V_IN_ of both chips calculated using the values obtained so far. From this graph, we can see that as C_S_ became smaller, ΔC_Smin_ also became smaller, and when C_S_ was sufficiently small, ΔC_Smin_ became constant and almost equal to the capacitance detection lower limit. Therefore, the maximum capacitance detection precision of Chip A was 70 zF when V_IN_ was 20 V and 5 zF when V_IN_ was 300 V, and that of Chip B was 100 zF when V_IN_ was 20 V and 8 zF when V_IN_ was 300 V. The plotted range at each V_IN_ corresponded to the detectable capacitance range. The lower limit of the capacitance detection range was determined by the chip noise level, while the upper limit was determined by the signal range of the chip or the distance and electro permittivity between the detection electrode and the measurement target, whichever was smaller.

Next, consider the distance detection precision. As shown in Figure 12a, C_S_ could be seen as a composite capacitance C_sensor_ from the detection electrode to the chip surface, which was calculated from the passivation film thickness, and capacitance C_ext._ from the chip surface to the object. The distance detection precision Δ*x*_min_ was the minimum Δ*x* at which this distance change could be detected as a capacitance change when x increases by Δ*x*. This Δ*x*_min_ can be expressed as the following Equation (3).

(3) $$\Delta x_{\min}=\varepsilon_{0}\varepsilon_{r}S\left\{{\left(C_{S}-\frac{V_{\mathrm{RN}}{\left(C_{C}+C_{S}\right)}^{2}}{V_{\mathrm{RN}}\left(C_{C}+C_{S}\right)+C_{C}V_{\mathrm{IN}}}\right)}^{-1}-\frac{1}{C_{\mathrm{sensor}}}\right\}-x\;\left(C_{S}=\frac{C_{\mathrm{sensor}}\varepsilon_{0}\varepsilon_{r}S}{C_{\mathrm{sensor}}x+\varepsilon_{0}\varepsilon_{r}S}\right)$$

C_sensor_ is an intrinsic value determined by the structure of the chip. Substituting each value into this equation, the relation between *x* and Δ*x*_min_ for both chips is shown in Figure 12c. Here, V_IN_ was set to 5.2 V for Chip A and 59 V for Chip B so that the output signal of each chip was close to saturation when *x* was 0. As shown in this figure, the smaller the *x*, the higher the distance precision, and both chips could detect distance differences on the order of nanometer when *x* was less than 1 μm.

**Figure 13:**
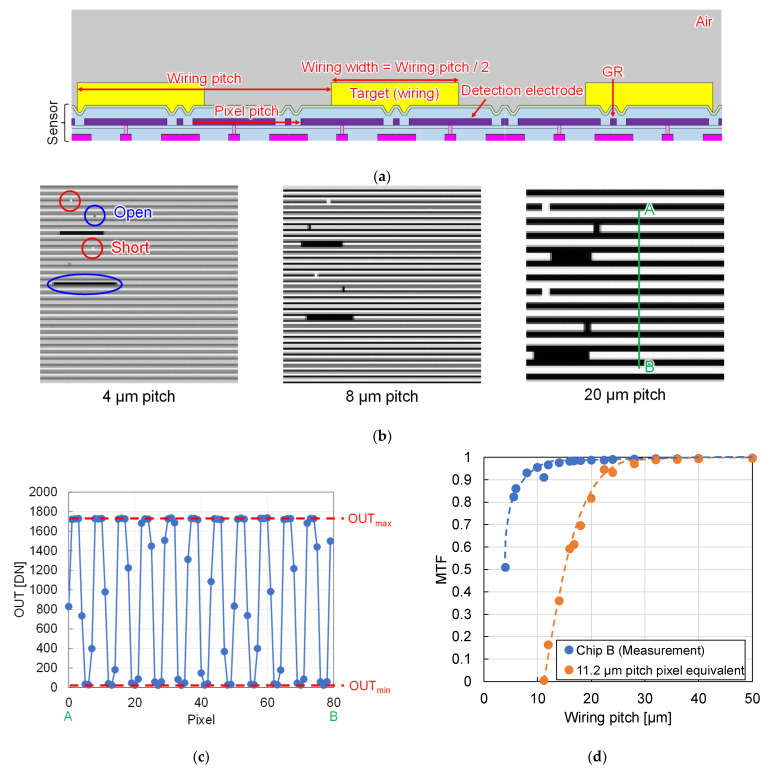
Resolution measurement of Chip B: (**a**) cross-sectional structure of the chip with metal wiring created on the surface; (**b**) captured images of the wiring for each pitch; (**c**) output signal for each pixel when 20 µm pitch wiring captured by Chip B; (**d**) MTF calculated from the measurement results.

Figure 13 shows the resolution measurement of Chip B. In order to calculate MTF, which is an index of resolution, TiN metal wires of 100 nm thickness having several different pitches were formed in the chip fabrication process as targets on the surface of Chip B as shown in Figure 13a, and images were captured by applying φC to these wires with a probe. The captured images are shown in Figure 13b. The visualization of intentionally designed open and short parts were confirmed even for the smallest 4 μm pitch wiring. For the 20 µm pitch wiring, the shape of the wiring was clearly captured. The output of pixels along with the line A-B in Figure 13b is shown in Figure 13c. From these results, MTF was calculated using the following Equation (4).

(4) $$\mathrm{MTF}=\frac{{\mathrm{OUT}}_{\max}-{\mathrm{OUT}}_{\min}}{{\mathrm{OUT}}_{\max}}$$

MTF at each wiring pitch was calculated from Equation (4) when the wiring pitch was varied between 4 and 50 μm, and the plotted MTF curve is shown in Figure 13d. The blue points were calculated from the measurement results using Chip B, and the orange points were calculated from the results of converting the measurement results of Chip B to a pixel pitch of 11.2 μm. With Chip B, MTF values of about 0.5, 0.9, and 0.99 were obtained when the wiring pitch was 4, 8, and 20 μm, respectively. These results show that the resolution of Chip B was greatly improved at a wiring pitch of 20 μm or less, and it was also resolved at 10 μm or less. This indicates that Chip B was useful for visualization of fine wiring and cells.

**Figure 14:**
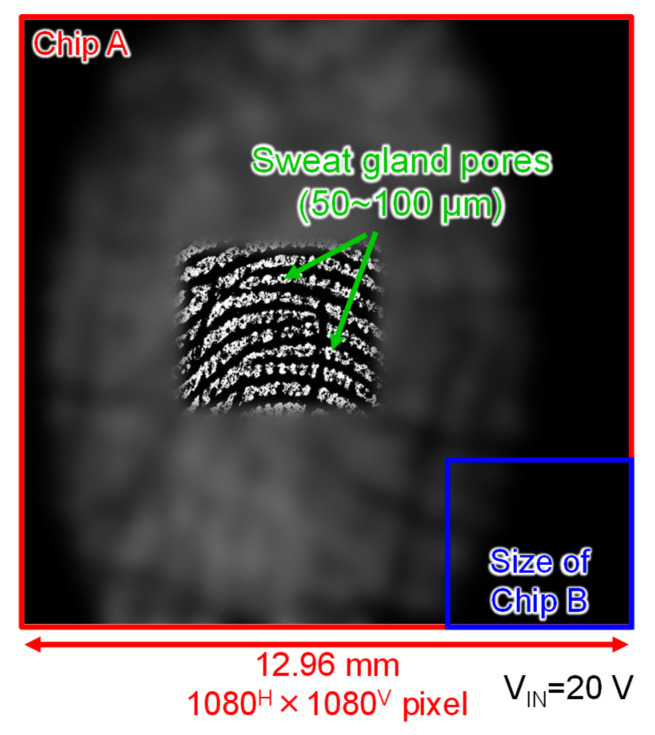
Captured image of a fingerprint by the large format Chip A (blurred for privacy).

Figure 14 shows a fingerprint image captured by Chip A (some areas are blurred to protect personal information). This image was captured by placing a finger in contact with Chip A, which was assembled in the manner shown in Figure 8c, and a probe applying φC with V_IN_ of 20 V was placed in contact with the body. The large pixel area allowed us to capture the entire fingerprint. Furthermore, sweat gland pores of several tens of micrometers in diameter were clearly captured.

**Figure 15:**
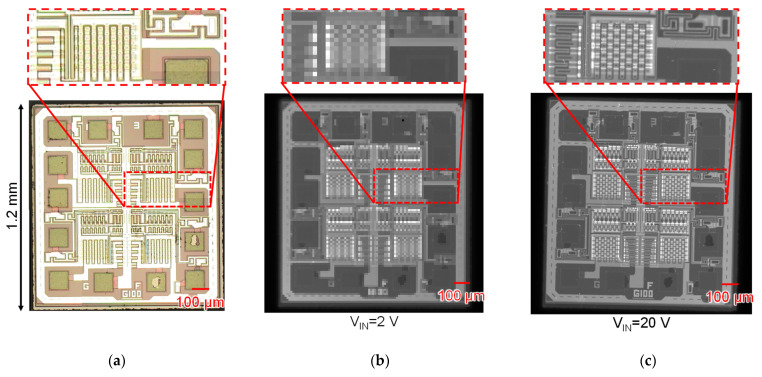
Captured images of a general-purpose logic IC (TC74HC02): (**a**) captured by optical image sensor using a microscope; (**b**) captured by Chip A; (**c**) captured by Chip B.

Figure 15 shows images of a general-purpose logic IC chip captured by an optical image sensor and Chip A and B. The target is a general-purpose logic IC chip; TC74HC02 fabricated by around 3 μm CMOS process. Image Figure 15a is a composite image of six images taken in each region using a 200× optical microscope and an optical image sensor. Furthermore, Figure 15b,c are images captured by contacting TC74HC02 with Chip A and B assembled by the method shown in Figure 8a, respectively, and applying φC by contacting the probe with the back surface of the target. In Chip B, the shape of transistors and wiring with a width of 10 μm or less could be clearly captured, indicating that the resolution has been improved compared to Chip A. Additionally, comparing Figure 15a,c, the capacitance image sensor was able to capture the shape of the wiring near the pad and the shape of the contacts and was able to visualize the difference in dielectric constant and unevenness.

**Figure 16:**
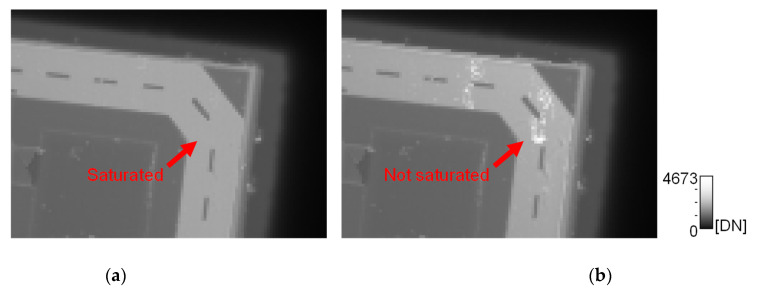
Comparison of images captured of a general-purpose IC chip with the sensor operating in normal mode and HDR mode: (**a**) Normal mode; (**b**) HDR mode.

Figure 16 shows enlarged images of the logic IC chip captured by Chip B operating in Normal mode and HDR mode. In Figure 16a, the image was captured in Normal mode with V_IN_ as a single 120 V. In Figure 16b, the image was captured in HDR mode with multiple V_IN_ applied at 15 V intervals below 120 V. Both images show the same signal range. In Figure 16a, the signal was saturated in the region with large capacitance, and it was impossible to distinguish the unevenness, but in Figure 16b, by using the signal with small V_IN_ in the region with large capacitance, it was possible to visualize the small unevenness without saturation. Thus, HDR mode expanded the range of detectable capacitance and enabled the detection of small capacitance differences while maintaining high SNR.

**Figure 17:**
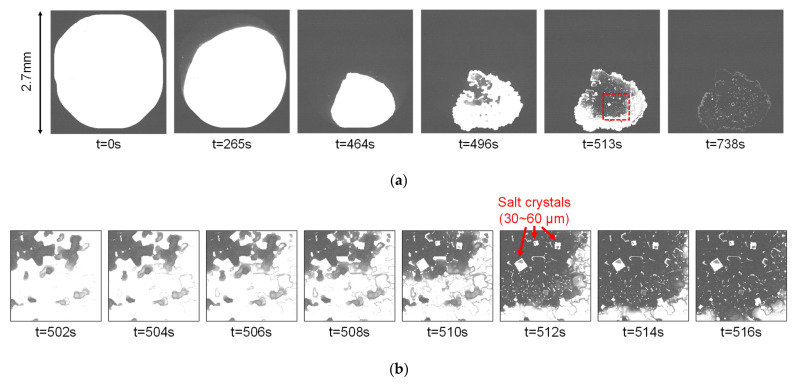
Captured images of a drop of saline solution on the sensor surface drying out as time advances captured by Chip B: (**a**) sample images; (**b**) closed-up images of red rectangle in (**a**).

Figure 17 shows images of the evaporation of saline solution dropped on the surface of Chip B assembled by the method shown in Figure 8b. Here, the images were captured by applying φC from the GR of a pixel in the chip, without using an external counter electrode. The images clearly show the evaporation of water with the passage of time and the eventual precipitation of salt crystals with a size of several tens of micrometers.

**Figure 18:**
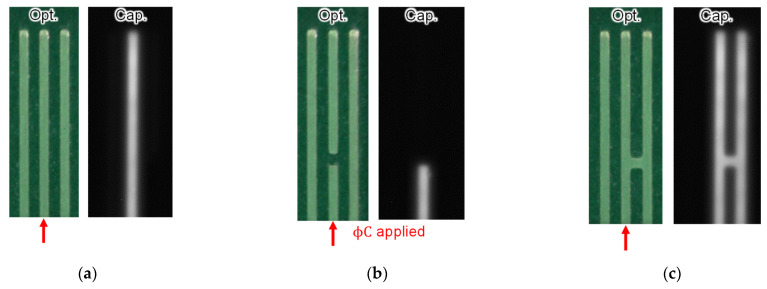
Captured images of printed circuit board wiring by optical image sensor and Chip A: (**a**) no defects; (**b**) open defect; (**c**) short defect. φC was applied only to the center wire indicated by the red arrows.

Figure 18 shows images of the wiring of a printed circuit board captured by an optical image sensor and a capacitance image sensor. The capacitance images were captured by Chip A, which was assembled in the manner shown in Figure 8c. φC was applied only to the center wire indicated by the red arrows in the figures. In Figure 18a, since there was no defect, only the center wire to which φC was applied appears in the capacitance image. In Figure 18b, the center wiring contained an open spot, and the wiring beyond it did not appear in the capacitance image. In Figure 18c, the center and right wires were shorted via a spot, so both wires appeared in the image. As shown above, in the capacitance image, only the wiring connected to the parts where φC was applied was visualized. Therefore, it was easy to identify the defect position, and the existence of the defect could be detected even if the defective part was outside the field of view.

**Figure 19:**
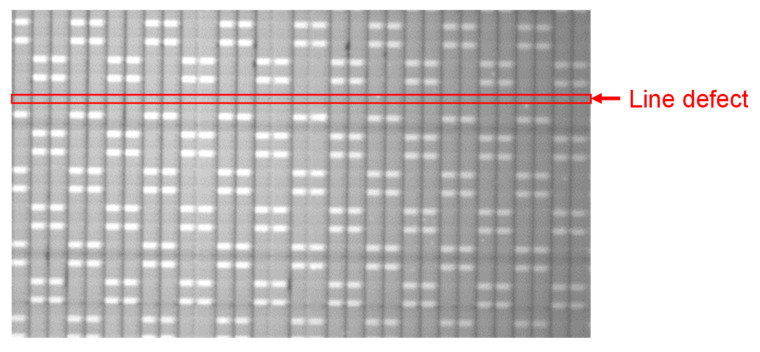
Captured image of flat panel display with a line defect by Chip A.

Figure 19 shows an image of a flat panel display (FPD) taken with Chip B. Since FPD used indium tin oxide, which has a high transmittance of visible light, as an electrode, it was difficult to inspect the wiring of FPD using an optical image sensor. Therefore, using a capacitance image sensor made it easier to visualize the electrical connections. In Figure 19, transparent electrodes with a pitch of 216 µm were lined up, and the electrode to which φC was applied appeared white. However, in the line indicated by the red arrow, no signal appeared, indicating a line defect. As shown above, the capacitance image sensor could be used for wiring inspection of FPD.

When detecting the capacitance of a large object, such as a wiring inspection, the sensor position was shifted one after another and then captured images while repeating the process (step-and-repeat). Therefore, by enlarging the sensor area, the number of shots could be reduced, and the inspection will be faster and more efficient.

Table 2 shows the performance of the developed chips. Compared to our previous work, Chip A achieved 10 times larger imaging area and a higher precision, and Chip B achieved 33 times higher resolution while maintaining the precision. Comparing the performances among Chips A and B, the input referred temporal random noise was smaller for Chip A, which resulted in a better detection precision. It was because chip A had a higher signal readout gain at the in-pixel SF and at the horizontal signal readout circuit using capacitive charge division operation. The temporal random noise of Chip B could be improved by increasing the signal readout gain by introducing column parallel gain amplifiers and ADC for instance.

**Figure 20:**
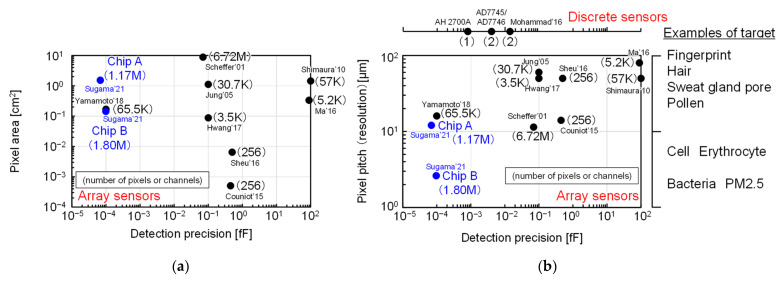
Benchmark comparison with other sensors [6,9,10,11,12,13,14,15,16,17,18,19,20,21,22,23,26]: (**a**) comparison of detection precision and pixel area; (**b**) comparison of detection precision and pixel pitch and examples of target.

Figure 20 shows the benchmarking of the developed proximity capacitance CMOS image sensors with other array and discrete capacitance sensors [6,9,10,11,12,13,14,15,16,17,18,19,20,21,22,23,26]. The developed sensors had a large pixel area and a higher resolution while achieving higher detection accuracy than those of other sensors.

## 4. Conclusions

Two high-precision proximity capacitance CMOS image sensors were presented in this paper. Both chips successfully achieved real-time proximity capacitance imaging with high precision. Using Chip A with a large pixel area, the measurement efficiency was improved especially for large measurement targets like flat panel displays. It was production-ready for wiring inspection applications. For Chip B, with high-resolution pixels, it was expected to be useful for visualization of microscopic objects such as living cells. The developed sensors could be utilized for high-efficiency measurement tools in manufacturing, biomedical, life science fields, and more.

## Figures and Tables

**Table 1:** Measured noise value of the two chips.

Noise	NC	Chip A	Chip B
Fixed pattern noise (input referred)	w/o	19.3 mV_rms_	16.4 mV_rms_
	w/	37.8 µV_rms_	137 µV_rms_
Temporal random noise (input referred)	w/o	1.29 mV_rms_	1.71 mV_rms_
	w/	267 µV_rms_ (w/o averaging), 25.2 µV_rms_ (100 frames average)	887 µV_rms_ (w/o averaging), 85.5 µV_rms_ (100 frames average)

**Table 2:** Performance summary of the developed sensors.

	Previous Work [17,18,19,20,21,22]	Chip A [21,22,23,25]	Chip B [23]
Process	1-Poly 5-Metal 0.18 µm CMOS		
Die Size	4800 µm^H^ × 4800 µm^V^	14,400 µm^H^ × 14,400 µm^V^	4800 µm^H^ × 4800 µm^V^
Pixel Area	4096 µm^H^ × 4096 µm^V^	12,960 µm^H^ × 12,960 µm^V^	3942.4 µm^H^ × 3584 µm^V^
number of Pixels	256^H^ × 256^V^	1080^H^ × 1080^V^	1408^H^ × 1280^V^
Pixel Pitch	16 µm^H^ × 16 µm^V^	12 µm^H^ × 12 µm^V^	2.8 µm^H^ × 2.8 µm^V^
Detection Electrode Size	12 µm^H^ × 12µm^V^	8.2 µm^H^ × 8.2 µm^V^	1.56 µm^H^ × 1.56 µm^V^
Frame Rate	60 fps	7 fps	8 fps
Sampling Frequency	20 MHz	20 MHz	20 MHz
Saturation Signal (input referred)	1.03 V	1.70 V	1.89 V
Temporal Random Noise (input referred)	321 µV_rms_ (w/o averaging), 55.1 µV_rms_ (100 frames average)	267 µV_rms_ (w/o averaging), 25.2 µV_rms_ (100 frames average)	887 µV_rms_ (w/o averaging), 85.5 µV_rms_ (100 frames average)
Maximum Detection Precision	1 × 10^−19^ F (V_IN_ = 20 V)	7 × 10^−20^ F (V_IN_ = 20 V), 5 × 10^−21^ F (V_IN_ = 300 V)	1 × 10^−19^ F (V_IN_ = 20 V), 8 × 10^−21^ F (V_IN_ = 300 V)

## Abbreviations

The following abbreviations and Parameters are used in this manuscript:

CMOS	Complementary Metal Oxide Semiconductor
a	Atto (10^−18^)
z	Zepto (10^−21^)
S/H	Sample and Hold
SF	Source Follower
HDR	High Dynamic Range
RN	Random Noise
FPN	Fixed Pattern Noise
GR	Guard Ring
GND	Ground
CIS	CMOS Image Sensor
TEM	Transmission Electron Microscope
AFE	Analog Front End
FPGA	Analog to Digital Converter
NC	Noise Cancelling
PTC	Photon Transfer Curve
f	Femto (10^−15^)
SNR	Signal to Noise Ratio
MTF	Modulation Transfer Function
IC	Integrated Circuit
FPD	Flat Panel Display
**Parameters**	
C_C_	Detection electrode capacitance
C_S_	Measurement capacitance
V_IN_	Input pulse amplitude
V_TH_	Transistor threshold voltage
V_OUTN_	Voltage of thermal noise and SF VTH variation
V_OUTS_	Voltage of signal including noise and variation
V_OUT_	Signal voltage after noise cancelling
G_SF_	SF amplifier gain
V_FPN_	Voltage of FPN
V_RN_	Voltage of RN
ΔC_Smin_	Capacitance detection precision
C_sensor_	Capacitance between detection electrode and chip surface
C_ext._	Capacitance between chip surface and counter electrode
x	Distance between chip surface and counter electrode
Δx_min_	Distance detection precision
ε_0_	Permittivity of vacuum
ε_r_	Relative permittivity
S	Detection electrode area
